# Fabrication of lithium niobate fork grating by laser-writing-induced selective chemical etching

**DOI:** 10.1515/nanoph-2021-0446

**Published:** 2021-01-05

**Authors:** Tianxin Wang, Xiaoyi Xu, Lei Yang, Shuo Yan, Xueli Hu, Xiaopeng Hu, Xiaomei Lu, Min Xiao, Yong Zhang

**Affiliations:** National Laboratory of Solid State Microstructures, College of Engineering and Applied Sciences, School of Physics, and Collaborative Innovation Center of Advanced Microstructures, Nanjing University, Nanjing, 210093, China; Department of Physics, University of Arkansas, Fayetteville, AR, 72701, USA

**Keywords:** chemical etching, femtosecond laser writing, fork grating, lithium niobate, vortex beam

## Abstract

We propose and experimentally demonstrate a laser-writing-induced selective chemical etching (LWISCE) technique for effective micro-fabrication of lithium niobate (LN) crystal. Laser writing of LN crystal produces negative domains and domain walls. Also, it causes local lattice defects, in which the etching rates are significantly increased in comparison to the original LN crystal. In experiment, we use the LWISCE technique to fabricate various fork gratings in an X-cut LN crystal for the generation of vortex beams. In comparison to etching an untreated X-cut LN crystal, the etching rates of the laser-writing-induced boundaries and the central laser-irradiated areas are enhanced by a factor of 26 and 16, respectively. The width and depth of fork grating structure can be precisely controlled by laser writing parameters. Our method provides an efficient mask-free micro-fabrication technique for LN crystal, which can be readily applied to other ferroelectric crystals such as lithium tantalate, potassium titanyl phosphate and barium calcium titanate.

## 1 Introduction

Lithium niobate (LN) has become an important material for photonic integrated circuits because of its excellent piezoelectric, acoustic-optic, electro-optic, and nonlinear optical characteristics. By using micro-fabrication methods, one can fabricate waveguides [1], [2], [3], [4], [5], micro-disks [2, 5, 6], and photonic crystals on an LN surface, which compose various functional devices such as electro-optic modulators [7], [8], [9], optical filters [10, 11], optical couplers [12, 13], and optical frequency converters [14, 15]. LN-based integrated devices show great potentials in optical communication [16], frequency comb generation [17, 18], nonlinear holography [19, 20], and quantum computation [21]. It is critical to develop efficient and precise micromachining techniques for LN crystal. Currently, the popular method of LN micro-fabrication starts from the production of a protective mask via lithographic techniques, such as, photolithography and electron-beam lithography. Then, the designed structures can be obtained through wet etching [22, 23], dry etching [1, 24], and chemomechanically polling [4, 5].

In this work, we report a mask-free laser-writing-induced selective chemical etching (LWISCE) technique to fabricate micro-structures on an LN surface. Generally, the etching rates of LN and other hard crystals are quite slow. The combination of femtosecond laser writing and chemical wet etching provides an effective way to produce micro-structures in hard crystals including sapphire and yttrium aluminum garnet [25, 26]. In these works, a high-energy laser pulse damages the crystalline of local area, which is then removed by wet etching. We propose a novel LWISCE mechanism for the fabrication of ferroelectric crystals including LN crystal. First, we use a near-infrared (NIR) femtosecond laser to pole the designed ferroelectric domain structure in LN crystal. The laser irradiation significantly increases the etching rate of LN surface. Then, selective wet etching is performed by utilizing different etching rates between the laser-irradiated areas, laser-nonirradiated areas and the laser-induced boundaries. Notably, the required laser pulse energy is much lower in comparison to previous laser-assisted etching techniques. In the experiment, we fabricate a high-quality fork-grating in an X-cut LN crystal for example.

## 2 Principle and methods

Laser poling of LN crystal is first realized by using ultraviolet light, in which strong absorption restricts the fabrication to a shallow surface. Now, the popular way is NIR femtosecond laser writing technique, which has been used to fabricate one-, two-, and three-dimensional domain structures in LN crystals [27, 28]. Because the LN crystal is transparent in NIR wavelength band, this technique is capable to perform deep-depth domain poling in LN crystal. Due to multiphoton absorption, the focused femtosecond laser pulses generate a local thermo-electric field, which is utilized to realize domain reversal in LN crystal. Such NIR femtosecond laser writing technique has shown unparalleled advantages in the fabrication of nonlinear photonic crystals especially with three-dimensional structures. In early studies, chemical etching is generally used to distinguish the positive and negative ferroelectric domains in LN crystals [28, 29]. Here, we show that the combination of laser poling and wet etching can realize efficient mask-free micro-fabrication on LN crystals.

**Figure 1:**
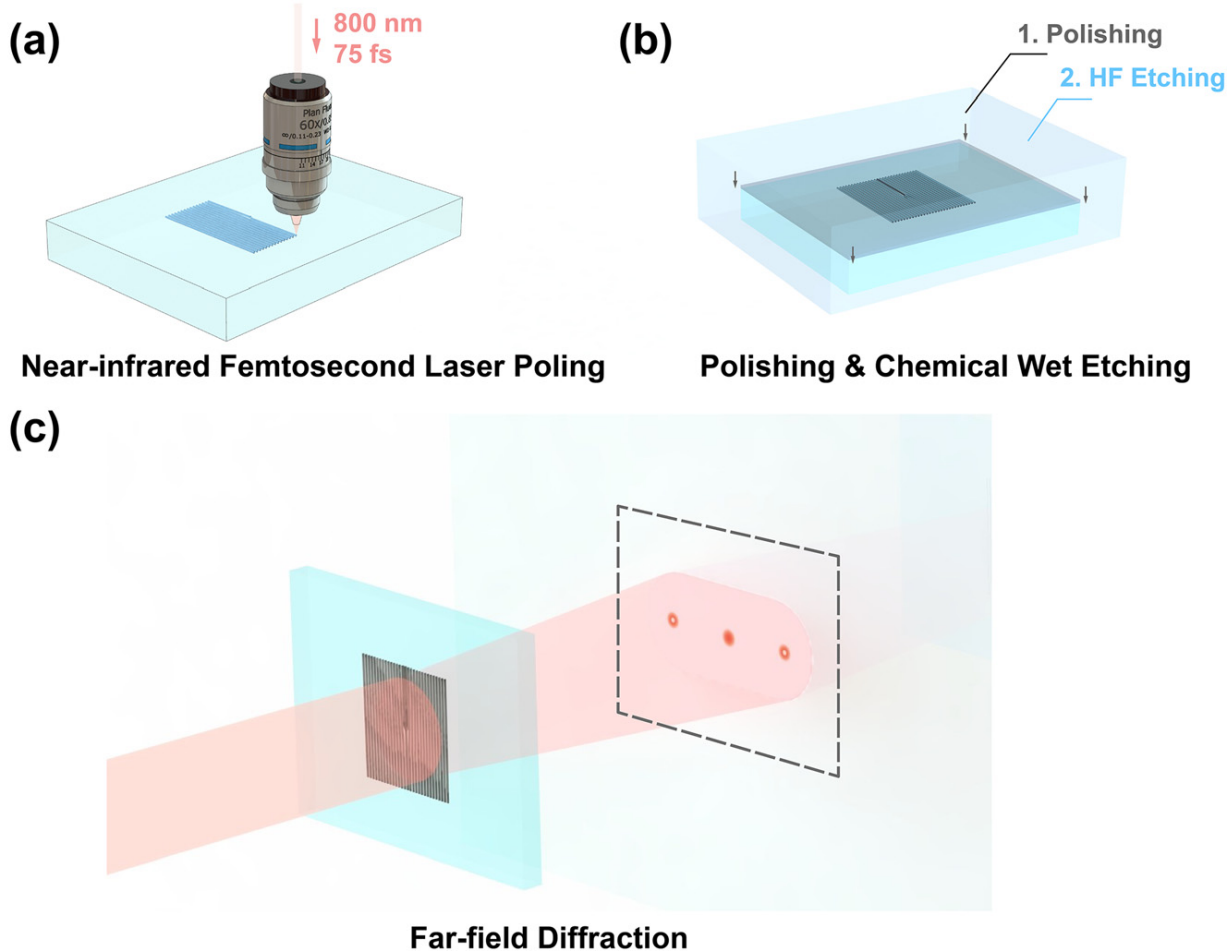
(a) The scheme of laser poling by using an oil immersion objective to focus the NIR laser beam. (b) The sample is first polished until the laser-poled area is exposed and is then etched in 40% HF solution. (c) We use the LWISCE technique to fabricate fork gratings with different *l* orders, which is tested by optical diffraction.

In this work, we use a tightly focused NIR laser to invert the domain structures in an LN crystal. A MgO-doped X-cut LN wafer is cut into pieces of 10 × 1 × 1 mm^3^ for laser poling. The laser source is a tunable femtosecond laser (Chameleon Vision-S, Coherent Co.), which works at an 800 nm wavelength, a 75 fs pulse width, and an 80 MHz repetition rate. A half-wave plate and a polarizing beam splitter are used to enable fine adjustment of the used laser power during the poling process. As shown in Figure 1a, after optical beam collimation, the NIR laser is tightly focused in the LN sample by an oil immersion objective (63×, N.A. = 1.4). The LN crystal is mounted on a nano-positioning piezo-stage, which is controlled by a Labview program to achieve dynamic motion control. To investigate the mechanism of LWISCE, we first fabricate a periodic domain structure with a 3 µm period at 30 µm depth under the surface of the X-cut LN crystal. The used pulse energy is 3 nJ in our experiment. Mechanical polishing is applied to thin the sample until the laser-poled area is exposed (Figure 1b). Then, the LN sample is etched in 40% HF aqueous solution. We record the surface structures of LN crystal at different etching stages. At the beginning and the end of each etching stage, the sample is treated in an ultrasonic bath for 20 min, which is used to achieve full contact between the sample and the HF aqueous solution and to remove the reaction products.

## 3 Results

**Figure 2:**
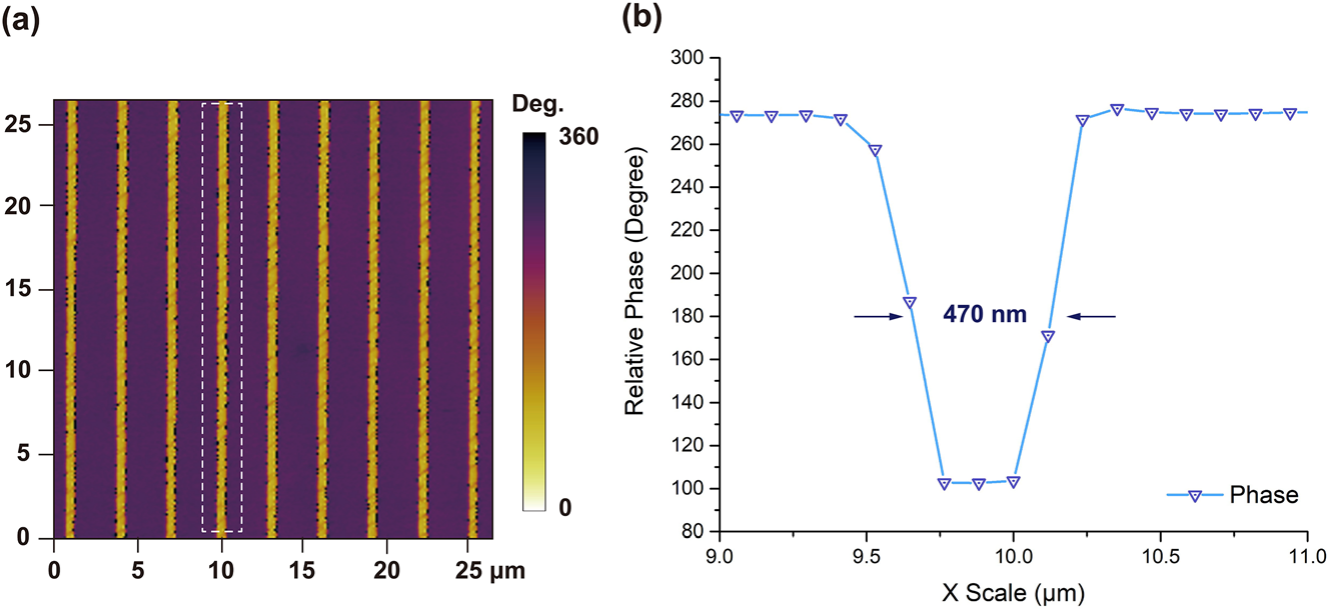
(a) The in-plane phase image after laser-poling. The curve in (b) is averaged across the area indicated by the dotted line in (a), which shows a 180° phase difference in the reversed domain. The width of the reversed domain is 470 nm.

Before the etching process, the effect of laser poling is first examined by using a piezo-response force microscopy (PFM). Domain-reversed structures are clearly observed in the in-plane phase image (Figure 2a). The cross-sectional view (Figure 2b) of the phase image shows a 180° reversed domain, which is well consistent with the antiparallel polarization directions of the positive and negative domains in LN crystal. The average full-width at half-maximum (FWHM) of the reversed domain is measured to be 470 nm.

**Figure 3:**
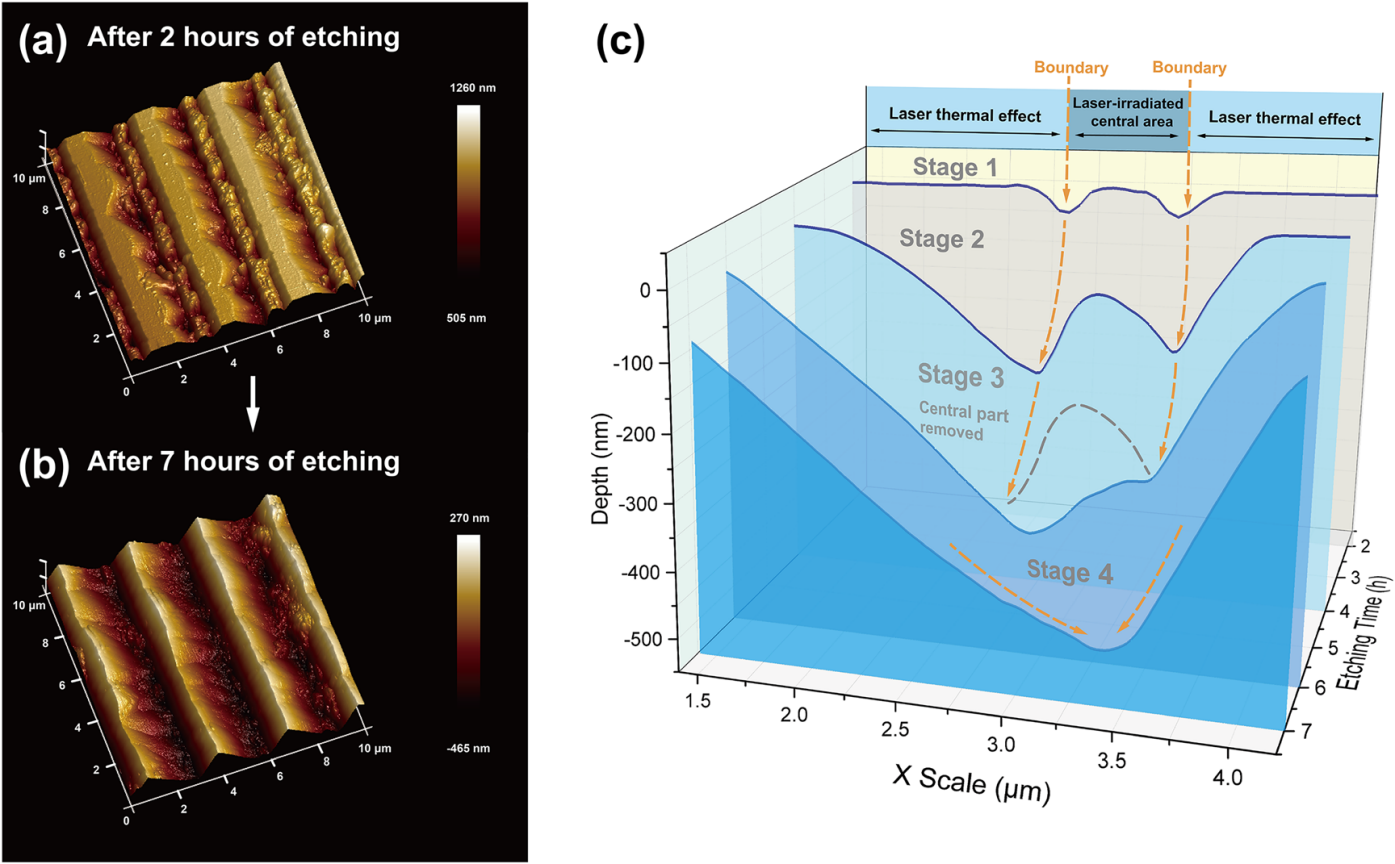
(a) and (b) are the surface morphologies after etching for 2 and 7 h, respectively. (c) shows the evolution of LN surface morphology after 2 h (stage 1), 4 h (stage 2), 6 h (stage 3), and 7 h (stage 4) etching. The gray dotted line in stage 3 indicates the removed laser-irradiated area.

The laser-poled LN crystal is then etched in 40% HF aqueous solution at room temperature. The entire etching process takes 7 h, which is divided into four stages. After each etching stage, we use atomic force microscopy (AFM) to scan the surface topography of the same laser-poled area to monitor the kinetic processing of LWISCE. Figure 3a and b shows the AFM images of the grating structures after 2 and 7 h, respectively. The etching process starts from the edge of the laser-poled area as shown in Figure 3a. After 7 h etching, the laser-poled area is completely removed and a grating structure with a V-shaped cross-section is obtained (Figure 3b).

Figure 3c shows the evolution of the LN surface morphology at each stage. These cross-sectional curves are averaged across the 10 × 10 μm^2^ area as shown in Figure 3a and b. In the first stage, the laser-poled LN sample is etched for 2 h. Shallow grooves with depths of tens of nanometers are formed, which locate at the surface of laser-induced boundaries. Clearly, the laser-induced boundary is the easiest to be etched, which open the initial etching channel. This can be attributed to that crystal lattice defects are severely distorted at these boundaries during the laser poling process. After etching the LN sample for another 2 h (i.e., at stage 2), the grooves become wider and their depths reach 220 nm. In the curve of stage 2, one can distinguish three laser-affected areas with various etching rates, which correspond to different interaction mechanisms. The etching rate at the boundaries is fastest. The etching rate in the central laser-irradiated areas comes the second. Note that the etching rates of the LN crystal within laser illumination are all increased in comparison to the area out of laser illumination. This can be attributed to the thermal accumulation under laser irradiation, which produces non-negligible stress [30]. After etching for 6 h (at stage 3), the etching channels are further extended close to the bottom of the laser-affected area. In comparison to stage 2, the central laser-irradiated areas (marked by a gray dotted line in Figure 3c) are almost removed at stage 3. Next, we clean the sample with Argon ions for 15 min and immerse it in 95% ethanol solution with an ultrasonic processing for 90 min. Then, the last 1 h etching process (stage 4) removes the residual area modulated by the NIR femtosecond laser.

The final depth of the groove after 7 h etching is measured to be 470 nm (Figure 3c). During the etching process, the opening of the groove in Figure 3c is continuously extended from 1 to 3 µm. As previously reported, the etching rate of an X-cut LN surface is typically below 3 nm h^−1^ [3]. In our experiment, the sample surface out of laser illumination remains optically flat after etching. Considering the etching property of X-cut LN crystals [31, 32], the defects at the laser-writing-induced boundaries plays a dominant role in enhancing the etching rate of LN surface. Thermal-accumulation-induced stress also has considerable contributions to the etching speed [26, 30]. By using the experimental data in stage 1 and stage 2, we calculate the etching rates to be 81 and 51 nm h^−1^ for the laser-induced boundaries and the central laser-irradiated areas, respectively, which is enhanced by a factor of 26 and 16 in comparison to etching an untreated X-cut LN crystal. This LWISCE technique provides a novel way for efficient mask-free fabrications of micro-structures on LN surface.

**Figure 4:**
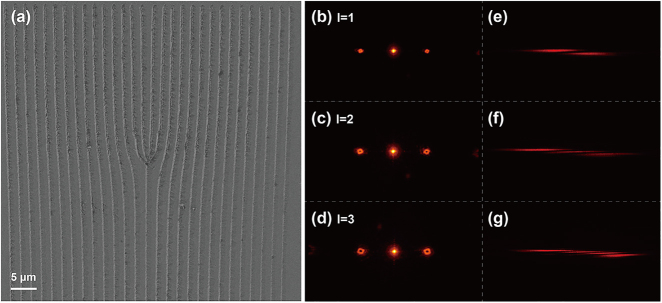
(a) SEM image of the *l* = 3 fork grating fabricated by LWISCE. (b)–(d) are the ±1-order diffraction patterns from *l* = 1, 2, and 3 fork-gratings. (e)–(g) are the transformed patterns after a cylindrical lens, in which the *l* value can be obtained by counting the number of dark strips.

Next, we use the LWISCE technique to fabricate various fork gratings for the generation of vortex beams. The vortex beam, which carries orbital angular momentum (OAM) of light, features a spiral wavefront and ring-shaped intensity distribution. Vortex beams have been widely applied in optical manipulation [33, 34], optical communications [35], precision measurement [36], holography [37, 38], nonlinear and quantum optics [39, 40]. In experiment, we fabricate three fork gratings with the topological charges of *l* = 1, 2, and 3 (see supplementary information for details). The total size of each fork grating is 60 × 60 μm^2^ and the period is 2 µm. Figure 4a shows a scanning electron microscopic (SEM) image of the *l* = 3 fork grating. The groove structure has a duty cycle of approximately 1:1, which guarantees the diffraction efficiency of the fork grating. The diffraction patterns are measured by using a 750 nm laser as shown in Figure 4b–d, in which the optical vortexes of different *l* orders can be clearly seen. The *l* order of each vortex beam is examined by using a cylindrical lens [41]. The dark strips in the transformed patterns in Figure 4e–g correspond to the values of *l*. We first measure the powers of +1 and −1 order OAM modes separately. Considering the reflection loss, the total diffraction efficiencies of ±1 order OAM modes are calculated to be 19.2, 17.8, and 15.6% for the fork-gratings of *l* = 1, 2, and 3, respectively.

## 4 Conclusions

We have demonstrated an LWISCE technique for efficient mask-free fabrication of LN crystal. After laser poling, the boundaries of laser-irradiated areas open up the initial etching channel. The etching rates at the laser-induced boundaries and the central laser-illuminated areas are greatly accelerated. To examine the performance of this method, we fabricate various fork gratings to generate vortex beams of different orders. Such LWISCE technique is capable to facilitate the micro-fabrications of high-quality integrated photonic devices in ferroelectric crystals including LN and lithium tantalate crystals.

## Supplementary Material

Supplementary Material
