# Supplementary material for: Fabrication of lithium niobate fork grating by laser-writing-induced selective chemical etching
Source: Nanophotonics. 2021 Jan 5;11(4):829–34. doi: 10.1515/nanoph-2021-0446 (PMC11501449; doi:10.1515/nanoph-2021-0446)
Supplement: Supplementary file 1 — Supplementary Material [file j_nanoph-2021-0446_suppl.docx]

**Supplementary Information**

1. **The design of fork gratings**

Based on the binary computer-generated-hologram theory^1^, the structure function can be written as:

$$f(x,y)=T\{\cos[G_{x}x-\arg(E_{vortex})]-\cos[\sin^{-1}amp(E_{vortex})]\}$$

Here, $E_{\text{vortex}}$ is the target beam, *G_x_* is the spatial frequency along *x* direction, and “*arg*” and “*amp*” represent the phase and amplitude of $E_{\text{vortex}}$, respectively. In order to generate a vortex beam, we set $amp\left(E_{vortex}\right)\text{=}1$ and define the function *T* as

$T(x)=\left\{\begin{matrix} a,\text{ }x\geq0 \\ b,\text{ }x<0 \end{matrix}\right.$,

where a and b correspond to the etched and unetched areas, respectively. Taking different spiral phases into Eq. 1.1, we can get the structures of fork gratings with *l* = 1, 2, and 3 (Fig. 1(a)-1(c)). The fabricated fork gratings are shown in Fig. 1(d)-1(f). The total size of each fork grating is 60 × 60 μm^2^ and the period is 2 µm.


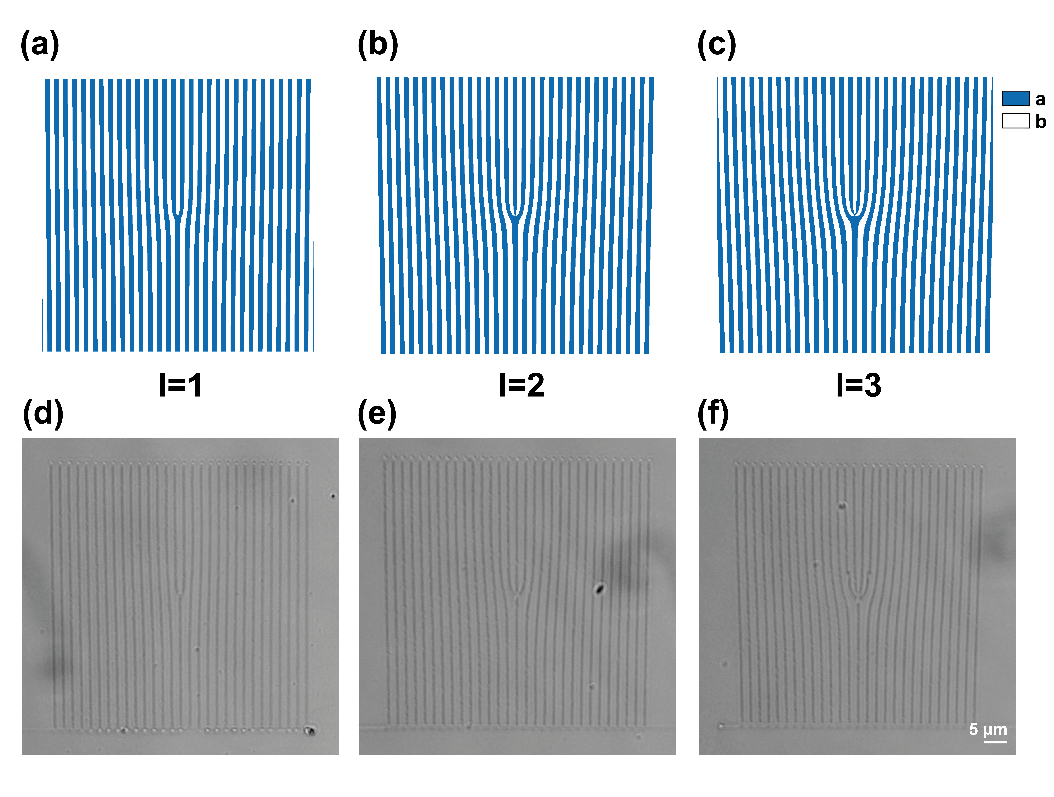


Figure 1. (a)-(c) show the designed fork gratings of different *l* orders. (d)-(f) are the microscopic images of the fork gratings.

**Reference**

1. Lee, W.-H. Binary computer-generated holograms. Applied Optics 18, 3661-3669 (1979).
